# Characteristics of Medical Evacuation by Train in Ukraine, 2022

**DOI:** 10.1001/jamanetworkopen.2023.19726

**Published:** 2023-06-23

**Authors:** Stig Walravens, Albina Zharkova, Anja De Weggheleire, Marie Burton, Jean-Clément Cabrol, James S. Lee

**Affiliations:** 1 Medical Department, Médecins Sans Frontières–Operational Centre Brussels, Brussels, Belgium; 2 Department of Emergency Medicine, Ghent University Hospital, Ghent, Belgium; 3 Operations Department, Médecins Sans Frontières–Operational Centre Brussels, Lviv, Ukraine; 4 Operations Department, Médecins Sans Frontières–Operational Centre Brussels, Brussels, Belgium; 5 Department of Critical Care Medicine, University of Alberta, Edmonton, Alberta, Canada

## Abstract

**Importance:**

The 2022 war in Ukraine severely affected access to health care for patients in the conflict-affected regions and limited options for medical evacuation. Air transport, a common method of medical evacuation in war zones, was unsafe due to the conflict of 2 modernized military forces that were in possession of aircraft and surface-to-air weapons; therefore, Médecins Sans Frontières, in collaboration with the Ukrainian railway company and Ukrainian health agencies, addressed this by initiating medical evacuation via medically customized trains.

**Objective:**

To describe the implementation of medical evacuation trains aimed at improving the access to health care for war-affected patients.

**Design, Setting, and Participants:**

This case series describes the remodeling of 2 trains used for medical evacuation in a conflict zone during the war in Ukraine. The study was conducted from March 30 to November 30, 2022. One train had minimal adjustments and could be rapidly deployed to address the most pressing humanitarian needs, while the other underwent major structural modifications to provide intensive care capacity. The report details the medical capabilities of the trains, the organization of referrals, and operational challenges encountered. Additionally, it includes a case series on the characteristics of patients transported in the initial 8 months, based on routinely collected programmatic descriptive data of all patients transported by the medical trains.

**Results:**

In 8 months, 2481 patients (male-female ratio, 1.07; male, 1136 [46%]; female 1058 [43%]; missing data, 287 [12%]; median age, 63 years [range, 0-98 years]) were evacuated from 11 cities near the Ukrainian conflict frontline to safer areas. Initially, the trains predominantly evacuated trauma patients, but over the course of the war, the patient characteristics changed with more medical and nonacute conditions, and fewer trauma patients. The main reason for entry into the intensive care unit train carriage was for close monitoring and observation, and the main interventions performed were primarily for respiratory failure.

**Conclusions and Relevance:**

The findings of this study suggest that medical evacuation in a war zone by converted trains is possible and can improve access to health care for war-affected patients. The presence of intensive care capacity on board allows for transport of more severely ill or injured individuals. However, the target population should not be limited to trauma patients, as health care institutions affected host a much broader population whose needs and urgency for evacuation may change over time.

## Key Points

**Question:**

How can medical evacuation trains be implemented in a war zone and what type of patients can be expected?

**Findings:**

This case series describes medical evacuation by train during the 2022 Ukraine conflict using 2 trains. Over 8 months, the medical trains made 74 journeys, evacuating 2481 patients from 11 cities close to the frontline; during this period, the most common type of patients transported changed from trauma-related casualties to patients with medical and nonacute reasons for referral.

**Meaning:**

The findings of this study suggest that medical evacuation by train can be feasible in a conflict zone with a preexisting railway network, if adapted to local conditions and an evolving patient population.

## Teaser

This case series describes the use of trains for evacuation of individuals with medical needs, including from ambulatory to intensive care, in Ukraine in 2022.

## Introduction

At the first anniversary of the 2022 war in Ukraine, a report of the office of the High Commissioner for Human Rights reported 21 580 civilian casualties of whom 8101 were killed, noting the actual figures could be considerably higher due to minimal information from areas most under siege.^1^ Access to safe and essential medical care near the frontline was severely compromised due to active weaponized combat and limited transportation options due to roadblocks, damaged roads, and collapsed bridges. Health care staff fled, hundreds of health care facilities were attacked, and the remaining functional medical services were overwhelmed with trauma patients.^2,3,4^ Moreover, the initial mass evacuations of affected areas often left older, sick, or institutionalized people behind without continuity of care, which sooner or later put them in a situation that required medical evacuation.^5,6,7^

Médecins Sans Frontières (MSF), an independent, medical humanitarian organization already present in Ukraine before the war, explored ways to relieve overcrowded hospitals and bring conflict-affected patients to safer regions. Air transport, the most frequently reported technique of medical evacuation in war zones,^8^ was unsafe due to the conflict of 2 modernized military forces possessing aircraft and surface-to-air weapons. Furthermore, the airspace was closed to civilian aircraft. Evacuation by road had inherent challenges due to long distances, frequent roadblocks, and greater need for human and material resources. However, Ukraine’s railway system was initially only minimally affected, allowing for the transportation of larger numbers of patients to safe areas. Minimal literature exists on this alternative evacuation modality. The last large-scale wartime medical evacuation by train took place during the Korean conflict of 1950 to 1953.^9^ However, more recently, train transport of patients with COVID-19 to intensive care units (ICUs) happened in France.^10^

This study describes a program of medical evacuation by train aimed at improving access to health care for war-affected patients. It reports on the remodeling of 2 trains for medical use, including 1 with ICU capacity, staffing, patient selection, train route selection, organization of referrals, and associated operational challenges. Additionally, it includes a case series on the characteristics of patients transported in the initial 8 months, need for referral, and the type of care provided.

### Program Description

#### Train Adaptations

The medical and logistic department of the MSF drafted feasibility, technical, and strategic plans, and consulted local stakeholders. In collaboration with the National Ukrainian railway company, 2 Ukrainian-made passenger trains were adapted to prepare them for medical transport. Remodeling was guided by previous experience with COVID-19 trains in France.^10^ However, aspects of war and the local context needed to be taken into consideration.

The basic medical train had minimal modifications and was launched to respond quickly to the most pressing humanitarian needs. It consisted of 2 sleeper carriages with modified partitions to allow stretchers to enter, 1 sleeper carriage for ambulatory patients with less severe conditions, and 1 staff carriage. Patients’ companions could stay in the upper bunk beds or in unoccupied lower beds. The train was equipped with medications and materials, oxygen concentrators, suctioning, and emergency equipment for intubation or chest drains. Up to 32 immobile patients and 27 ambulatory patients could be transferred.

While the basic train was put in use on March 31, 2022, construction of a more advanced medical train with ICU capacity was still under way (eFigure in Supplement 1). Extensive remodeling was performed, modifying the electrical system and stripping the interior to accommodate hospital beds and equipment. The train consisted of 8 carriages: 1 carriage with 5 ICU beds of which 2 could offer invasive mechanical ventilation, 2 carriages with 9 beds for nonambulatory patients, 1 regular sleeper carriage, and 1 carriage with beds and mattresses for ambulatory patients with nonsevere conditions and their companions. The 3 remaining carriages were for staff, medical stock, oxygen generators, and an uninterruptable power supply, which was needed to allow transport of ICU patients over long distances on an unreliable electrical railway grid. Exclusive use of oxygen cylinders was deliberately not considered as the train risked being stuck for long periods in case of damaged railway infrastructure and would carry a high explosive risk in a war zone. The oxygen-generating system could provide oxygen at a rate of 30 L/min to each ICU bed, while the 2 ICU beds with mechanical ventilation were able to provide 60 L/min and constant pressure between 2.8 and 6.0 bars needed for the ventilator. The structural changes, technical modifications, and equipment installations of the advanced medical train took 23 days, and first deployment happened on April 24, 2022. Minor adjustments continued after train deployment. Air conditioning was installed in the spring, while the oxygen carriage was equipped with electrical heating and thermally insulated oxygen pipes for winter temperatures to preserve the functioning of oxygen generators, which highlights the importance of being able to further modify the trains after deployment to react to encountered or anticipated problems.

#### Train Staff

Apart from a few MSF international staff, it was mainly local individuals who were recruited to staff the train. These were physicians with ICU experience, nurses, nurse aides, technical logisticians, and a translator. The number of staff increased over time, allowing more rotations and more nurses per patient. International staff consisted of a project coordinator, an emergency/ICU physician, a nurse manager, and once the advanced train was launched, an ICU nurse. Local staff were trained to gradually take over some of these positions. Ukrainian Railway staff remained in charge of routine train functioning and movements.

#### Patient and Train Route Selection

The number and severity of patients referred needed to be considered when accepting a transfer. Patients required a certain clinical stability for transport as a 1-way trip lasted, on average, 21 hours and advanced diagnostic or therapeutic options on the train were limited. Initially, the capacities of the train were not well known to the referring institutions. As a result, the number and/or acuity of the patients was often higher than anticipated. Because the train’s medical staff was responsible for the health of patients once they were on board, urgent medical and ethical decisions whether to accept patients for transport often had to be made on the platform. Acceptance meant exposing the patient to a long train journey with constraints in space, ICU capacity, and access to life-saving surgery, while refusal meant sending the patient back to the referring hospital where conditions were not well known. The implementation of referral guidelines, which were distributed to the Ministry of Health of Ukraine, regional departments of health and referring hospitals (eAppendix 1 and eAppendix 2 in Supplement 1) improved this process considerably. In general, the basic medical train accepted patients of all ages in stable condition and without ICU requirement. Patients receiving oxygen were allowed if their baseline usage was less than 5 L/min. With deployment of the advanced train, more critically ill patients could be accepted, and criteria were extended to patients receiving vasopressors or mechanical ventilation.

Initially, the Ministry of Health of Ukraine assisted in identifying patients for transport and functioned as liaison with the different referring and accepting departments of health, hospitals, and institutions. They collected information on potential candidates, arranged transport to and from the railway stations, and helped organize appropriate distribution of patients to different receiving health care facilities. Subsequently, MSF established the position of medical liaison, a local MSF physician with experience on the evacuation train, based in the eastern city of Dnipro. The liaison collaborated closely with the Ministry of Health representative in establishing patient lists, verifying the suitability of every proposed patient transfer, checking bed availability on the train, and improving communications. After 3 months, the medical liaison was well established and dealt directly with the various regional departments of health for both referral and acceptance of patients.

Once health care facilities most in need were identified, a train route itinerary was planned. Together with the railway company, the closest accessible railway station was sought, security situation assessed, and timetable set. Initially, train routes were set only 1 or 2 days before departure, allowing maximum adaptability to respond to the most pressing humanitarian needs. Nevertheless, this system was strenuous for the railway company to fit it into a busy railway network and for referring hospitals to identify and prepare their patients for transfer on time. Gradually, the planning of train routes evolved into a mixed system that also included preplanned and repetitive visits to certain regions. This helped large referral hospitals to plan ahead and allowed for the evacuation of institutions such as an orphanage and psychiatric institutions. The medical liaison identified institutions requiring urgent evacuation and institutions that could accept the specific type of patients being evacuated.

#### Organization of the Referral

The train mostly left the base city of Lviv in the afternoon and planned to arrive the next day around midday. Embarkment of patients needed to be carefully planned as time on the platform was restricted due to safety risks. To illustrate why, less than a day after the basic train evacuated 40 patients from Kramatorsk, multiple rockets hit the same platform, killing at least 50 individuals.^11^

During the outward journey, staff prepared the train for the arrival of patients. Physicians assessed the list of planned patients and anticipated the severity and type of care needed to allocate beds and resources properly. Shortly before arrival of the train, patients were transferred from the health facilities to the railway station. Ideally, ambulances queued up at the platform on arrival. Nevertheless, the security situation and limited availability of ambulances led to situations in which ambulances were delayed or had to leave for a new assignment before the train arrived. This resulted in major differences in the number of personnel who were present on the platform to help transfer patients onto the train, and sometimes forced the train staff to make quick shifts in embarkment strategy in order to allow for a timely departure. The experience of the first embarkments led to a standardized embarkment strategy aimed at minimizing time loss in assigning each patient to the most optimal bed position while still being able to adapt to unexpected situations (eAppendix 3 and eAppendix 4 in Supplement 1). A physician coordinated the embarkment and triaged every patient before entry in the train, while a nurse assigned them to a suitable bed within the carriages. A color-coded tag was pinned to each patient at triage to signal their assigned carriage, which had a corresponding-colored flag at the entrance. The most critical patients were positioned in beds near the nursing station in the basic train or in the ICU carriage in the advanced train. Patients who were ambulatory with less need of nursing care were placed in a regular sleeper carriage. Nonambulatory patients were carried in on a stretcher by ambulance personnel or by MSF staff in their absence. As space is restricted in a carriage and limited to 1 entry, stretchers needed to be carried in one-by-one or in series, pressuring time management. The advanced train was more spacious to maneuver inside but carried fewer nonambulatory patients overall. Furthermore, its length of 8 carriages meant carriages were further apart and could complicate accessibility from some shorter platforms.

Once on the train, a thorough assessment of each patient took place, and the necessary medical treatment was continued to allow safe and comfortable transport. All staff were needed in these initial busy hours to settle patients and establish a care plan before staff could switch to a shift system in the evening. Specialized treatments relevant for a long transport context were sometimes used, such as peripheral nerve blocks. These were performed to avoid respiratory complications in selected patients with high opioid requirements due to aggravated pain of their injuries by a bumpy, moving train. Diagnostic and therapeutic options remained limited on the basic medical train and, on rare occasions if patients’ conditions deteriorated, this resulted in an unplanned stop at a railway station en route for an urgent patient transfer to the closest appropriate health care facility. Implementation of the advanced medical train ensured better care of the patients with deteriorating status and allowed for the transport of more critically ill patients, promoting it as the main train used for evacuations.

Patient care onboard the trains also differed from regular prehospital transport care in other aspects. Many patients had been denied access to health services for a long time, and with the increase in medical and social cases, the staff was frequently engaged in primary health care, such as restarting treatment for chronic medical comorbidities. Mental health was a major concern for many patients and necessitated training staff on how to assist these patients and how to cope with the stories they heard themselves. Staff actively identified specific social or mental health needs in patients, which were then followed up by MSF social workers after arrival. They mostly provided assistance on aspects of shelter, links to family, charity and volunteer organizations, social support, or mental health services.

In preparation of arrival the next morning, the definite number, severity, type, and mobility of patients needed to be communicated to the Ministry of Health or regional departments of health to allow planning of transport to the receiving hospitals. These institutions in the relatively safer western regions of the country had less disrupted health care systems. Nevertheless, a sustained influx of newly evacuated patients could soon overwhelm their capacity.^12^ Trauma patients who needed further surgery or long-term rehabilitation could often be transferred to hospitals abroad facilitated by the European Union Civil Protection Mechanism.^13^ In contrast, patients with other medical and social care needs remained predominantly in Ukraine and were consciously dispersed across several western provinces to redistribute the patient load.

After disembarkment, an MSF logistical team came on board to remove garbage, replace dirty linen, and restock medical material and drugs. The latter were kept in standardized boxes and replaced entirely after each trip, not to lose time restocking. A cleaning team cleaned and disinfected the carriage and equipment. A few hours later, the train often left again.

## Methods

All patients had demographic and clinical case management data collected as part of routine operations. These routine programmatic data were anonymized and analyzed for the first 8 months of train deployment (March 30 to November 30, 2022). Patient characteristics and clinical features were summarized using medians and ranges for continuous variables and percentages for categorical variables. Where applicable, this report follows the Strengthening the Reporting of Observational Studies in Epidemiology (STROBE) reporting guideline for observational studies. This manuscript fulfilled the exemption criteria set by the MSF Ethics Review Board for a posteriori analysis of routinely collected clinical data and thus did not require review by the MSF Ethics Review Board. It was conducted with permission of the medical director of MSF Operational Centre Brussels.

## Results

In 8 months, the MSF trains made 74 journeys, evacuating 2481 patients from 11 cities close to the frontline. The median age of transported patients was 63 (range, 0-98) years, and slightly more male patients were evacuated (male-female ratio, 1.07; male, 1136 [46%]; female 1058 [43%]; missing data, 287 [12%]). A total of 1098 patients (44%) were from a vulnerable population with a nonacute condition as the medical reason for referral, 721 patients (29%) had a recent or semirecent trauma, 605 patients (24%) had a medical condition, 55 patients (2%) had a nontraumatic surgical condition, and 2 patients (<0.1%) had an obstetric condition. General information on train evacuations and patient demographic characteristics can be found in Table 1, and Table 2 lists the various reasons for referral. The Figure shows the evolution of the type of reason for referral of the patients. No patient died on the train, 2421 (98%) reached the final destination, and 5 patients (0.2%) left the train on their own accord; data were missing for 55 patients (2%). Seven patients were transferred from the train before arrival at destination due to medical deterioration; however, this was not captured in the routine data collection.

**Table 1.** General Information on Train Evacuations and Patient Demographic Characteristics

Variable	Basic medical train	Advanced medical train	Both trains
No. of evacuations (%)	13 (18)	61 (82)	74 (100)
No. of patients (%)	483 (19)	1998 (81)	2481 (100)
Male/female ratio^a^	0.88	1.12	1.07
Median age, y (range)^b^	63 (1-94)	63 (0-98)	63 (0-98)

^a^ Missing data of 287 patients.

^b^ Missing data of 642 patients.

**Table 2.** Reasons for Referral

Variable	No. (%)		
	Patients of non-ICU carriages	Patients of ICU carriage	All patients
Medical condition^a^	548 (24)	57 (30)	605 (24)
Nonacute condition^b^	1098 (48)	0 (0)	1098 (44)
Obstetrics	1 (<1)	1 (1)	2 (<1)
Surgical, nontrauma (%)	52 (2)	3 (2)	55 (2)
Trauma			
Accidental^c^	65 (3)	13 (7)	78 (3)
Violent^d^	513 (22)	115 (61)	628 (25)
Unclassified	15 (1)	0	15 (1)
Total	2292 (100)	189 (100)	2481 (100)

Abbreviation: ICU, intensive care unit.

^a^ Medical condition: acute illness; not part of the surgical, trauma, or obstetrics groups.

^b^ Nonacute condition: vulnerable population in need of evacuation, but without capabilities to take regular passenger train due to underlying condition or extremes of age.

^c^ Trauma, accidental: burns, falls, traffic, or other accidents.

^d^ Trauma, violent: bomb blast, gunshot, edge weapon, landmine, shrapnel, projectile, shelling, or other violence.

**Figure.**
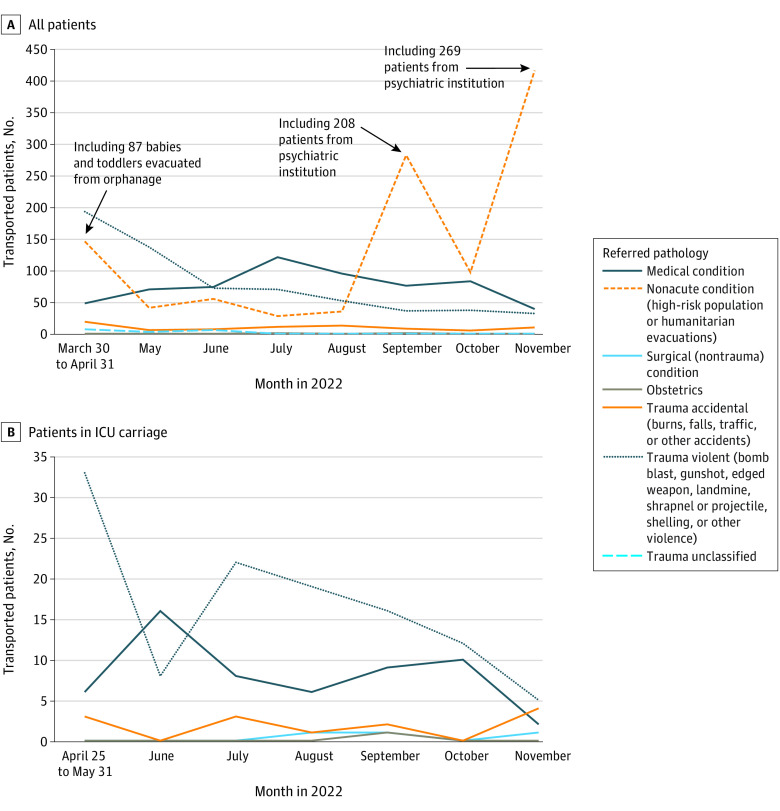
Reasons for Referral by Medical Train Over Time Data shown for all patients (A) and patients in the intensive care unit (ICU) carriage (B).

The main reasons for transport in the ICU carriage (n = 189) were for acute respiratory failure (16%), hemodynamic instability or shock (13%), altered mental status (21%), and metabolic disturbances (5%). The remainder was for close observation and monitoring (45%). The main interventions reported inside the ICU train carriage were oxygen administration (n = 41), invasive mechanical ventilation (n = 23), tracheostomy care (n = 18), central line management (n = 33), vasopressors (n = 5), and peripheral nerve blocks (n = 2).

## Discussion

At the beginning of the conflict, direct trauma cases constituted most of the evacuated patients, but their proportion diminished over time. In the ICU train carriage, trauma continued to be the main reason for referral over time, although a similar decrease occurred. In part, this could be attributed to the stabilization of the frontline with fewer direct civilian casualties.^1,14^ Furthermore, other state and nonstate actors started to provide long-distance transport of trauma patients, and hospitals in conflict-affected regions increased their capacity to care for ICU patients. The shift toward vulnerable populations with chronic comorbidities, older individuals, and institutionalized patients could partly be explained by the impairment of health care facilities and services disrupting the continuity of care.^5,6^ However, the reduced number of trauma patients also led to more proactive identification of these vulnerable groups.

Throughout the war, referring hospitals still seemed to be capable of performing primary surgery for trauma and referred, as the guidelines instructed, mostly stabilized patients for less-urgent secondary or tertiary surgery, or for rehabilitation. This is reflected as the main reason for ICU train carriage entry was for close observation and monitoring (45%) and in the minimal need of vasopressor (3%) treatment. Nevertheless, many of these patients were still critically ill, as 41 patients needed oxygen (22%) and 23 patients (12%) were receiving mechanical ventilation.

### Limitations

There are inherent limitations to our reported patient data, which were collected primarily for operational purposes. These included missing or incorrect data and variability in clinical care, all of which limit the validity of the results.

## Conclusions

This program report and case series describes how evacuation by means of medicalized trains improved access to health care in war-torn Ukraine. Rapid commissioning of a train was achieved by adapting existing carriages for patient transport. However, to increase the level of care provided with ICU capacity, an extensive structural remodeling was performed. Referral guidelines, a medical liaison, and a flexible embarkment strategy all helped in correct patient selection and improved patient safety on board. In addition, trauma cases constituted most transports at the beginning; however, the disruption of patient care continuity caused a shift toward more medical and nonacute reasons for referral over time and increased the need for primary health, psychological, and social care.

## Notes

Supplement 1. **eFigure.** Detailed Diagram of the Set-up of the Carriages for the Advanced Medical Train **eAppendix 1.** Information Leaflet Basic Medical Evacuation Train MSF—Version April 2022 **eAppendix 2.** Information Leaflet Advanced Medical Evacuation Train MSF—Version May 2022 **eAppendix 3.** Embarkment of Basic Medical Evacuation Train MSF—Version April 2022 **eAppendix 4.** Embarkment of Advanced Medical Evacuation Train MSF—Version May 2022

Supplement 2.
Data Sharing Statement
