# Supplementary material for: Characteristics of Medical Evacuation by Train in Ukraine, 2022
Source: JAMA Netw Open. 2023 Jun 23;6(6):e2319726. doi: 10.1001/jamanetworkopen.2023.19726 (PMC10290241; doi:10.1001/jamanetworkopen.2023.19726)
Supplement: Supplement 1. — eFigure. Detailed Diagram of the Set-up of the Carriages for the Advanced Medical Train eAppendix 1. Information Leaflet Basic Medical Evacuation Train MSF—Version April 2022 eAppendix 2. Information Leaflet Advanced Medical Evacuation Train MSF—Version May 2022 eAppendix 3. Embarkment of Basic Medical Evacuation Train MSF—Version April 2022 eAppendix 4. Embarkment of Advanced Medical Evacuation Train MSF—Version May 2022 [file jamanetwopen-e2319726-s001.pdf]

Supplementary Online Content

Walravens S, Zharkova A, De Weggheleire A, Burton M, Cabrol JC, Lee JS. Characteristics of medical evacuation by train in Ukraine, 2022. *JAMA Netw Open*. 2023;6(6):e2319726. doi:10.1001/jamanetworkopen.2023.19726

eFigure 1. eFigure 1. Detailed Diagram of the Set-up of the Carriages for the Advanced Medical Train

eAppendix 1. Information Leaflet Basic Medical Evacuation Train MSF—Version April 2022

eAppendix 2. Information Leaflet Advanced Medical Evacuation Train MSF—Version May 2022

eAppendix 3. Embarkment of Basic Medical Evacuation Train MSF—Version April 2022

eAppendix 4. Embarkment of Advanced Medical Evacuation Train MSF—Version May 2022

This supplementary material has been provided by the authors to give readers additional information about their work.

© 2023 Walravens S et al. *JAMA Network Open.*

eFigure. Detailed diagram of the set-up of the carriages for the advanced medical train

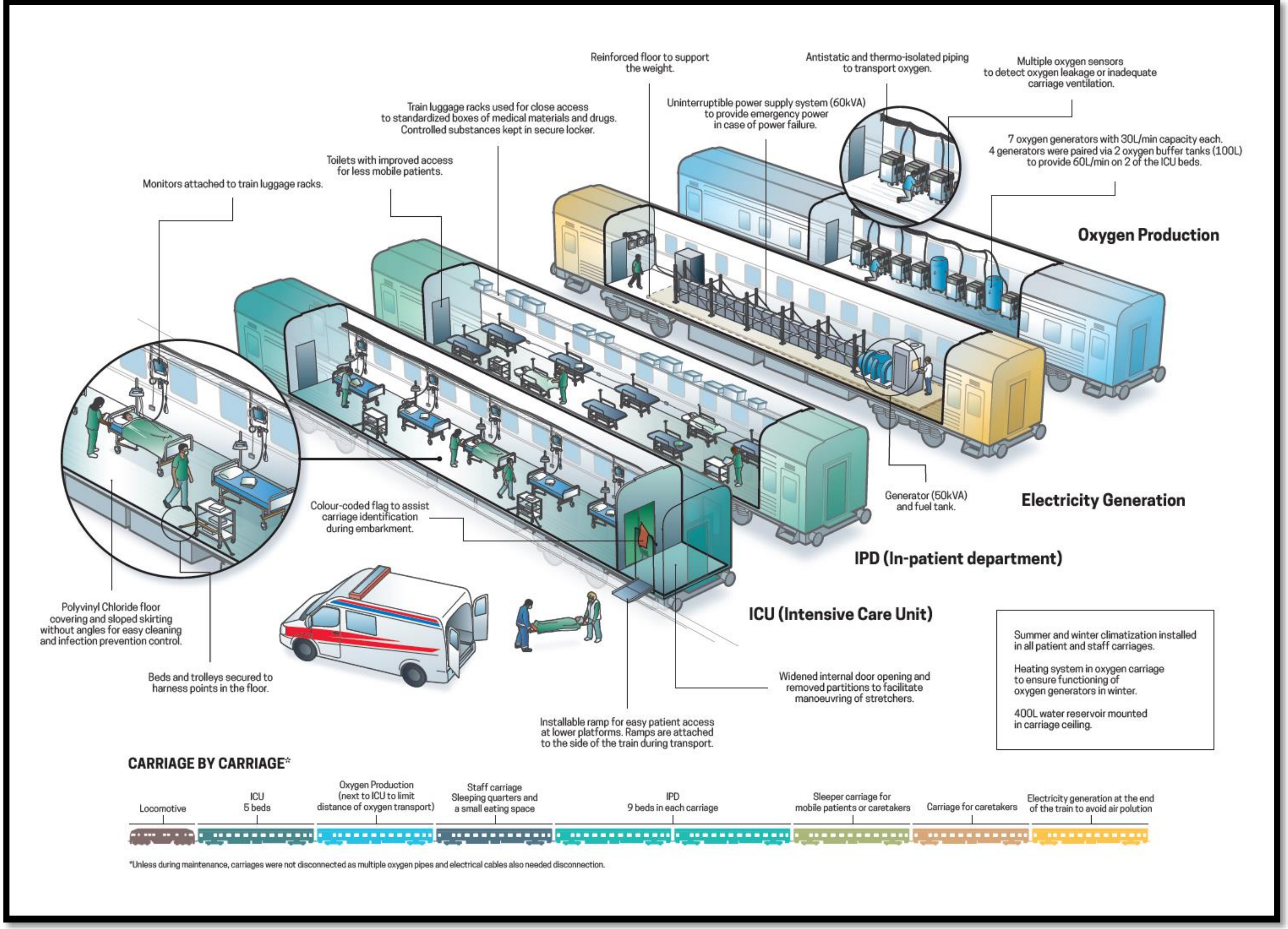

© 2023 Walravens S et al. *JAMA Network Open.*

eAppendix 1. Information leaflet basic medical evacuation train MSF – Version April 2022

MSF Train Project

MSF has started a project to evacuate patients from hospitals that are close to the conflict and/or are overwhelmed due to the influx of wounded or loss of own medical staff. These patients are transported to hospitals further away from the conflict with capacity to care for these patients. Currently MSF has a 4-wagon train running for cardiorespiratory stable, non-ventilated patients. In the near future MSF hopes to introduce a new advanced train capable of transporting patients that need higher levels of medical care. The following info only applies to the first basic evacuation train.

Capacity

- 1 set of 4 wagons (UKR model) for transfers of patients.
    - 2 wagons with capacity of **32 lying** positions
        - 26 single beds and 3 double beds (can be used for only 1 patient if needed e.g. in case of bilateral external fixators.)
        - 20 top bunk beds for mobile care takers
    - 1 regular train wagon for walking patients who need minimal nursing care for **27 patients**. An extra 27 patients or caretakers can be transported if possible to take place on top bed bunk)
    - 1 wagon for staff

Type of patients
The train can take patients of all ages. Our main target group are those who cannot evacuate by themselves, need nursing care and will need further care or secondary interventions in a hospital once evacuated.
We only try to take stable patients without need for a high level of intensive medical care. These are patients who are expected to remain in the same clinical condition during the next 24 hours of transport, who do not need urgent surgery or 1 on 1 intensive nursing care, and who are not intubated or on vasopressor therapy. IV fluids, intermittent IV drugs, oxygen therapy and aspiration can be provided on the train. A patient on oxygen therapy can be transported if he or she is saturating well on less than 5 litres of oxygen per minute, and this has been stable or improving in the last 48 hours. As there are only 3 oxygen concentrators, the amount of these patients on oxygen is limited.

MSF will not check the status of patients (soldier or civilian) at the train. No weapons, military clothes or military personnel are allowed in the MSF train.

Train schedule
If feasible, MSF tries to keep the train continuously operational 24/7. Moving between the West and the East of the country with a minimum of interruptions. The main axis of travel is between Lviv and Dnipro. This trajectory will deviate according to requests by hospitals in need of evacuations if security and accessibility allows.

Transfer procedure:
To request transport of patients, the MSF liaison officer for transfers can be called on [redacted] or mailed on [redacted].
A request should be made by the hospital director or director of health. If possible, a request should group multiple patients together before contact. The MSF liaison officer will then evaluate if the

© 2023 Walravens S et al. *JAMA Network Open.*

patients are within our transport criteria by phone or by visit to the hospital. Information the MSF liaison officer will need is:

- Number of patients, number of patients who can walk, number of patients who can only be transported horizontally (on stretcher), number of caregivers.
- Main previous medical history
- Summary of current diagnosis, received major treatments during admission and current clinical condition (cardiorespiratory stability)
- Specific needs during transport
- Specific needs for accepting hospital
- Consent of the patients that they are to be transferred to a hospital/institution in L`vivs`ka or Ivano-Frankivska region.

Next the MSF liaison officer will check if the patients can be transferred on the next passage of the train, or a planning will be made when the train can be sent to the closest safe railway station. Please contact the MSF liaison officer again immediately in case the situation would change or a patient becomes instable.

Transfer of the patient to the railway station remains the responsibility of the referring hospital. If MSF is present on the location, support can be given based on their capacity.

Contact with accepting hospitals will generally be done by MSF, unless specific care is required for the patients. Transfer from the train to the accepting hospitals (e.g. Lviv, Ivano-Frankivsk,…) will be arranged by the accepting hospitals.

After the transfer, MSF will not transport people back to the initial places. MSF cannot take the responsibility of ensuring complete trip safety due to current situation or circumstances beyond MSF's control, nor can they take responsibility for further care or evolution of the clinical condition of the patient in the accepting hospitals.

What to provide to the referred patient?

- Complete medical file with summary and further treatment plan.
- Contact information of relatives – please also contact relatives about transfer.
- Please provide patient with specific drugs needed for the duration of transport (e.g. IV antibiotics, anticonvulsants, insulin, …) if possible.
- Please apply fresh bandages, stoma bags or diapers before transfer if possible.

Food, drinks and blankets on the train will be provided by MSF.

Проєкт «Лікарів без кордонів»: Евакуаційний потяг

«Лікарі без кордонів» - MSF - розпочали проєкт з евакуації пацієнтів із лікарень, які знаходяться поблизу зони конфлікту та/або перевантажені через приплив поранених або втрату власного медичного персоналу. Ці пацієнти транспортуються до лікарень, що знаходяться далі від зони конфлікту, з можливістю догляду за цими пацієнтами. Наразі MSF має 4-вагонний потяг для кардіореспіраторно-стабільних пацієнтів, які не потребують штучної вентиляції легень. Найближчим часом MSF сподівається представити новий удосконалений потяг, здатний перевозити пацієнтів, які потребують більш високого рівня медичної допомоги. Наведена нижче інформація стосується лише першого базового евакуаційного потягу.

© 2023 Walravens S et al. *JAMA Network Open.*

Можливості/Характеристики:

• сукупно 4 вагони (українського виробника) для перевезення пацієнтів.

o 2 вагони місткістю **32 лежачих** місця

- 26 одномісних ліжок і 3 двомісні ліжка (використовуються лише для 1 пацієнта, якщо існує потреба широкого ліжка через, наприклад, наявність двосторонніх зовнішніх фіксаторів).
- 20 двоярусних ліжок для мобільного персоналу з догляду за пацієнтами

o 1 звичайний вагон для **27** пацієнтів, які пересуваються самостійно та потребують мінімального медсестринського догляду. Додаткових 27 пацієнтів або осіб, які доглядають за ними, можна перевезти за можливості на двоярусних ліжках)

o 1 вагон для персоналу

Категорія пацієнтів

Потяг може перевозити пацієнтів усіх вікових категорій. Наша основна цільова група – це ті, хто не може евакуюватися самостійно, потребує медсестринського догляду та потребуватиме подальшого догляду або вторинного втручання в лікарні після евакуації.

Ми намагаємося приймати лише стабільних пацієнтів без необхідності високого рівня інтенсивної медичної допомоги. Це пацієнти, які, як очікується, залишаться в тому ж клінічному стані протягом наступних 24 годин транспортування, які не потребують термінового хірургічного втручання або інтенсивного медсестринського догляду 1 на 1, а також які не проходять інтубацію або не отримують вазопресорну терапію. На базі потягу можна застосовувати рідини для внутрішньовенного введення, періодичні внутрішньовенні препарати, надавати кисневу терапію та застосовувати аспіраційні системи. Пацієнта на кисневій терапії можна транспортувати, якщо він або вона добре насичується киснем в обсязі до 5 літрів на хвилину, і така сатурація стабільно зберігається або покращується протягом останніх 48 годин. Оскільки в наявності є лише 3 кисневих концентратори, кількість таких пацієнтів для кисневої підтримки обмежена.

MSF не перевірятиме статус пацієнтів (військовий чи цивільний), проте у потязі заборонені зброя, військовий одяг або військовий супровід.

Розклад руху

За можливості, MSF намагається забезпечити безперервну роботу потягу в форматі 24/7.
Переміщення між зсходом і сходом країни відбувається з мінімумом перерв.
Головна вісь руху – між Львовом і Дніпром. Ця траєкторія, в залежності від запитів лікарень, які потребують евакуації й з урахуванням критеріїв безпеки та можливостей доступу може змінюватись.

Алгоритм транспортування:

Щоб замовити транспортування пацієнтів, до відповідальної особи MSF можна зателефонувати за номером [redacted] або надіслати поштою за адресою [redacted]

Запит має подати директор лікарні або керівник департаменту охорони здоров'я. За можливості, запит має містити також попередньо складений перелік пацієнтів по групам для надання такого готового переліку під час телефонного контакту. Після цього координатор MSF оцінить, чи відповідають пацієнти нашим критеріям транспортування по телефону або під час візиту до лікарні.

Інформація, яка знадобиться відповідальній особі MSF:

- Кількість пацієнтів, кількість пацієнтів, які можуть ходити, кількість пацієнтів, яких ожна транспортувати лише горизонтально (на ношах), кількість осіб, які доглядають за ними.

- Основна попередня історія хвороби
- Зведення поточного діагнозу, основних методів лікування під час госпіталізації та оточного клінічного стану (кардіореспіраторна стабільність)
- Специфічні потреби під час транспортування
- Специфічні потреби для прийому в лікарню
- Згода пацієнтів на переведення до лікарні/установи у Львівській або Івано-Франківській області.

Далі відповідальний лікар MSF перевірить, чи можна перевезти пацієнтів при наступному проходженні потяга, або буде сплановано, коли потяг можна буде відправити до найближчої безпечної залізничної станції. Будь ласка, негайно зв'яжіться з відповідальною особою MSF, якщо ситуація зміниться або пацієнт стане нестабільним.

Перевезення пацієнта на залізничний вокзал залишається обов'язком лікарні, яка направляє пацієнта для транспортування. Якщо інші проекти MSF присутні на місці, підтримка може бути надана залежно від їх можливостей.

Контакти з лікарнями, які приймають пацієнтів, зазвичай здійснюють MSF, якщо пацієнтам не потрібен особливий догляд. Трансфер з поїзда до лікарень-приймачів (наприклад, Львів, Івано-Франківськ,…) організовують лікарні-приймачі.

Після передачі пацієнтів приймаючим лікарням, MSF не здійснює доставку пацієнтів назад до місця відправки. MSF не можуть взяти на себе відповідальність за забезпечення повної безпеки поїздки через поточну ситуацію або обставини, які не залежать від MSF, а також не можуть взяти на себе відповідальність за подальший догляд або розвиток клінічного стану пацієнта в лікарнях, які приймають пацієнта після передачі їм.

Що необхідно мати при собі пацієнтам, яких направили для транспортування потягом?

- Повну медичну карту з висновками (резюме) та подальшим планом лікування.
- Контактну інформацію родичів – також звертайтеся до родичів щодо переведення.
- Будь ласка, надайте пацієнту конкретні препарати, необхідні на час транспортування (наприклад, внутрішньовенні антибіотики, протисудомні засоби, інсулін, …), якщо це можливо.
- Будь ласка, накладіть свіжі бинти, забезпечте мішки для стоми або пелюшки перед транспортуванням, якщо це можливо.

Їжу, напої та ковдри при транспортування потягом забезпечить MSF.

© 2023 Walravens S et al. *JAMA Network Open.*

eAppendix 2. Information leaflet advanced medical evacuation train MSF – Version May 2022

MSF Train Project – Advanced Medical Evacuation Train

MSF has started a project to evacuate patients from hospitals that are close to the conflict and/or are overwhelmed due to the influx of wounded or loss of own medical staff. These patients are transported to hospitals further away from the conflict with capacity to care for these patients. MSF has currently 2 evacuation trains running. One 4-wagon train running for non-intubated hemodynamically stable patients. A second 8-wagon train capable of transporting stabilized high care critically injured patients with the aim to decrease patient load for hospitals to better accommodate new incoming patients. The following document only applies to this second advanced medical evacuation train.

Capacity

- 1 set of 8 wagons (UKR model) for transfers of patients.
  - 1 ICU with capacity of 5 ICU beds.
  - 2 IPD wagons with capacity of 18 beds in total for medium care who can be managed with intermittent monitoring
  - 1 wagon with 12 low beds and 12 top bunk beds that can be used by caretakers or by walking patients without need of nursing care.
  - 1 wagon for staff
  - 3 wagons for generator, oxygen production, medical stock and a polyvalent wagon.

Type of patients

The train transports two groups of patients.

- ICU patients of more than 1 year old that can benefit from evacuation for further specialised care and treatment in the destination hospital or/and to help free up ICU bed capacity in the referring hospital.
- Regular patients who cannot evacuate by themselves, need nursing care and will need further care or secondary interventions in a hospital once evacuated.

Referred patients can be mechanically ventilated , on oxygen therapy or on a low dose of vasopressors if they have reached a clinical stable situation. The number of patients on mechanical ventilation is limited to 2. There is continuous monitoring without invasive blood pressure measurement available for 5 patients and intermittent monitoring for the remainder of patients. Patients at risk of needing emergency surgical treatment within the next 24 hours will not be accepted.

MSF will not check the status of patients (soldier or civilian) at the train. No weapons, military clothes or military escorts are allowed in the MSF train.

Train schedule

In the beginning the MSF advanced medical evacuation train will only be running on demand if an evacuation of sufficient ICU patients (e.g. 5 ICU patients, 2 ICU patients and 10 medium care patients,….) is requested. When demand would be high this could change in the future to a continuous schedule between the East and the West of the country.

© 2023 Walravens S et al. *JAMA Network Open.*

Due to the length of the train, railway accessibility, the security situation and the time required to transfer complex patients, we are not capable of sending the train to every railway station in the country. MSF will evaluate at every request if the nearest railway station is feasible. Otherwise MSF will propose the closest feasible railway station for the transfer of the patients.

Transfer procedure:
To request transport of patients, the MSF liaison officer for transfers can be called on [redacted] or mailed on [redacted].
A request should be made by the hospital director or director of health. If possible, a request should group multiple patients together before contact. The MSF liaison officer will then evaluate if the patients are within our transport criteria by phone or by visit to the hospital. Information the MSF liaison officer will need is:

- Number of patients
- Main previous medical history
- Summary of current diagnosis, received major treatments during admission and current clinical condition (cardiorespiratory stability)
- Specific needs during transport (mechanical ventilation, oxygen, vasopressors, suctioning, …)
- Present catheters (Central venous line, urinary catheter,…) and drains (chest drain, abdominal drains,…)
- Specific requirements for accepting hospital
- Consent of the patients that they are to be transferred to a hospital/institution in L`vivs`ka or Ivano-Frankivska region.
- Number of accompanying caregivers or of patients who can walk or do not need nursing care (extra wagon)

Next the MSF team will plan the route and the date for the advanced medical evacuation train. Please contact the MSF liaison officer again immediately in case the situation would change, a patient becomes too unstable for transfer or no longer requires evacuation.

Transfer of the patient to the railway station remains the responsibility of the referring hospital. If MSF is present on the location, support can be given based on their capacity.

Contact with receiving hospitals will generally be done by MSF, unless specific care is required for the patients. Transfer from the train to the accepting hospitals (e.g. L'viv, Ivano-Frankivsk,…) will be arranged by the receiving hospitals.

After the transfer, MSF will not transport people back to their place of origin. MSF cannot take the responsibility of ensuring complete trip safety due to current situation or circumstances beyond MSF's control, nor can they take responsibility for further care or evolution of the clinical condition of the patient in the accepting hospitals.

What to provide to the referred patient?

- Complete medical file with summary and further treatment plan.
- Contact information of relatives – please also contact relatives about transfer.
- Provide patient with specific drugs needed for the duration of transport (e.g. IV antibiotics, vasopressors, anticonvulsants, insulin, …).
- Apply fresh bandages, stoma bags or diapers before transfer if possible.

Food, drinks and blankets on the train will be provided by MSF.

© 2023 Walravens S et al. *JAMA Network Open.*

Проект поїзда MSF – Спеціалізований медичний евакуаційний поїзд

Лікарі без кордонів (MSF) розпочали проект з евакуації пацієнтів з лікарень, що розташовані біля зони бойових дій та/або перевантажені через наплив поранених або втрату власного медичного персоналу. Пацієнти транспортуються до лікарень у безпечніших регіонах, що мають змогу надати необхідну допомогу хворим. Наразі курсують 2 евакуаційні поїзди. Один рухомий склад з 4 вагонів призначений для гемодинамічно стабільних пацієнтів без інтубації. Другий потяг із 8 вагонів перевозить стабілізованих пацієнтів з важкими травмами, які потребують інтенсивної терапії, з метою зменшення навантаження на лікарні, аби ті могли забезпечити кращі умови для нових пацієнтів. Цей документ стосується лише другого спеціалізованого медичного евакуаційного поїзда.

Місткість

- 1 рухомий склад з 8 вагонів (українська модель) для перевезення пацієнтів.
    - 1 вагон-реанімація на 5 реанімаційних ліжок
    - 2 вагони-стаціонари на 18 ліжок для інтенсивної терапії, де здійснюється періодичний моніторинг стану пацієнтів
    - 1 вагон з 12 нижніми та 12 верхніми полицями, де можуть перебувати доглядачі або пацієнти, що можуть ходити і не потебують нагляду медсестри
    - 1 вагон для персоналу
    - 3 вагони для генератора, кисневого обладнання, медичного складу та мультифункціональний вагон

Типи пацієнтів

Потяг перевозить дві групи пацієнтів.
- Пацієнти у відділенні інтенсивної терапії віком від 1 року, яким може бути потрібна евакуація для подальшого спеціалізованого догляду та лікування в лікарні у місці призначення та/або для того, щоб допомогти звільнити ліжка у відділеннях інтенсивної терапії в лікарніях, що направляють пацієнтів.
- Звичайні пацієнти, які не можуть евакуюватися самостійно, потребують медсестринського догляду та потребуватимуть подальшого догляду або додаткового втручання в лікарні після евакуації.

Направлені пацієнти можуть отримувати штучну вентиляцію легень, кисневу терапію або низькі дози вазопресорів, якщо вони досягли клінічної стабільності. Кількість пацієнтів на штучній вентиляції легенів обмежена (2). Для 5 пацієнтів реанімаційного вагону доступний безперервний моніторинг без інвазивного вимірювання артеріального тиску, а для решти пацієнтів – періодичний моніторинг.
Пацієнти з ризиком потреби екстреного хірургічного лікування протягом наступних 24 годин не приймаються до транспортування.

MSF не перевірятиме статус пацієнтів (військовий чи цивільний), проте у потязі заборонені зброя та військовий супровід.

Розклад руху

На початку, спеціалізований медичний евакуаційний потяг MSF здійснюватиме рейси згідно запитів та за умови достатньої кількості пацієнтів (наприклад, 5 пацієнтів реанімації, 2 пацієнти

© 2023 Walravens S et al. *JAMA Network Open.*

реанімації та 10 пацієнтів стаціонарних відділень тощо). Якщо у майбутньому попит зросте, потяг курсуватиме безперервно між заходом та сходом країни.

Через довжину поїзда, доступність залізничних шляхів, ситуацію з безпекою та час, необхідний для перевезення складних пацієнтів, ми не маємо змоги відправити поїзд на кожну залізничну станцію країни. Розглядаючи кожен запит, MSF оцінюватиме можливість евакуації з найближчої станції. В інакшому випадку MSF запропонує іншу найближчу станцію для перевезення пацієнтів.

Процедура перевезення:

Запити на транспортування пацієнтів можна подавати відповідальній особі MSF за номером [redacted] або надсилати електронною поштою за адресою [redacted]

Запит подається директором лікарні або директором департаменту охорони здоров'я і , якщо це можливо, вже має включати всіх пацієнтів, яких потрібно евакуювати. Координатор MSF оцінює, у телефонному режимі чи під час особистого візиту до лікарні, чи відповідають пацієнти нашим транспортним вимогам.
Інформація, яка знадобиться відповідальній особі MSF:
- Кількість пацієнтів
- Основна попередня історія хвороби
- Опис поточного діагнозу, отриманого основного лікування під час госпіталізації та поточного клінічного стану (серцево-легенева стабільність)
- Особливі потреби під час транспортування (штучна вентиляція легень, кисень, судинозвужуючі, відсмоктування тощо)
- Наявні катетери (центральний венозний катетер, сечовий катетер тощо) та дренажні системи (дренаж грудної клітини, дренаж черевної порожнини тощо)
- Особливі вимоги до приймаючої установи
- Згода пацієнтів на їх переведення до лікарні/установи у Львівській або Івано-Франківській області.
- Кількість супроводжуючих піклувальників або пацієнтів, які можуть ходити або не потребують медсестринського догляду (окремий вагон)

Далі команда MSF планує маршрут та дату рейсу спеціалізованого медичного евакуаційного поїзда. Будь ласка, зв'яжіться з відповідальним лікарем MSF негайно, якщо ситуація зміниться, стан пацієнта стане занадто нестабільним для транспортування або якщо пацієнт більше не потребуватиме евакуації.

Лікарня, що направляє пацієнтів, несе відповідальність за їх транспортування до залізничної станції. Якщо працівники MSF присутні у населеному пункті, де відбуватиметься евакуація, вони можуть надати посильну допомогу.

MSF тримає зв'язок з лікарнями, що приймають пацієнтів, якщо тільки останні не потребують особливої медичної допомоги. Трансфер від потяга здійснюється приймаючими установами (наприклад, у Львові, Івано-Франківську тощо).

Після транспортування MSF не перевозить пацієнтів назад до їхніх регіонів. Зважаючи на поточну ситуацію та надзвичайні обставини, що не підконтрольні MSF, організація не може гарантувати повну безпеку рейсів, а також не бере відповідальність за подальше лікування чи розвиток клінічного стану пацієнта в приймаючих лікарнях.

© 2023 Walravens S et al. *JAMA Network Open.*

Що підготувати для відправлення пацієнтів?

- Заповніть медичну карту з описом та подальшим планом лікування.
- Вкажіть контакти родичів, а також попередьте їх про перевезення
- Забезпечте пацієнта специфічними препаратами, необхідними під час рейсу (наприклад, внутрішньосудинні антибіотики, судинозвужуючі, протисудомні засоби, інсулін тощо).
- За можливості накладіть свіжі пов'язки, калозбирачі або підгузки до відправлення.

Їжа, напої та ковдри в поїзді забезпечуються MSF.

© 2023 Walravens S et al. *JAMA Network Open.*

eAppendix 3. Embarkment basic medical evacuation train MSF – Version April 2022

Embarkment of basic medicalized train

Introduction

The importance of a structured and fast embarkment of the train is essential to give the optimal care to our patients and to ensure maximal safety. *Structured* to separate the more severe patients from the less severe, to facilitate nursing care and to optimize patient comfort. *Fast* to remain within the planned time schedule and to minimize the time exposed on the platform in volatile areas.

Although we plan every embarkment before arrival, difficult and unexpected situation are likely to occur. The most standard situation is where all ambulances are lined up and the clinical status of every patient is known. Deviations of this situation that have happened or can happen are ambulance coming one by one, busses with non-mobile victims, patients left behind without healthcare workers present, more patients or more severe patients than anticipated, patients who are too unstable for transfer, abortion or speeding up of embarkation due to security issues,…

The standard plan or Plan A is described below. Whenever a different situation occurs it is needed to adapt each time to the situation and wherever needed the correct team members need to be informed.
In case there is no ambulance staff present to transfer the patients into the train, we switch to Plan B in which a large part of the MSF staff in the wagons is send outside to carry the patients on stretchers and wheelchairs into the wagons.

Plan A - Zones and roles

Before every embarkment of patients, a meeting will be called by the Project Coordinator and/or the expat ER MD to announce the estimated number and severity of patients and the anticipated situation in the railway station. Subsequently, the plan for the embarkment will be shared and all staff will be reminded of their role. Planned deviations of the standard plan can also be announced.

Triage

- Who
  - National staff MD
  - With support of expat ER MD if needed
- What
  - Assessment of situation for safety and plan of action. Adapt plan where needed and communicate this with the team.
    - Announce switch to PLAN B by radio in case extra helpers for transfer of patients are needed.
  - If present, introduce yourself to representative of all patients to explain plan
  - Define zones for triage and for allocation
  - Regulation of entry and exit of ambulances. Ensure smooth flow
  - Separation of wagon 1 and 2 patients from wagon 3 patients
    - Wagon 3 patients or GREEN patients are patients who have some mobility and can transfer themselves to the toilet, need minimal medical attention or nursing care and can take their own medication.

© 2023 Walravens S et al. *JAMA Network Open.*

    - These patients are referred to wagon 3 by foot or by wheelchair. The responsible of wagon 3 can be contacted by radio to send a wheelchair with helper
  - Wagon 1 (RED) or wagon 2 (YELLOW) are patients who need to be transferred by stretcher, are not mobile, need intermittent or continuous nursing care, or need help in taking medications.
    - These patients are referred on a stretcher to the allocator in front of these 2 wagons who will further allocate a bed.
    - A note is given to each of these patients indicating the following if needed
      - Front (critical patients to be allocated to the front of the wagon as these are closer to the nursing post).
      - Area of injury - side of drains or external fixators (to help allocate these patients to a single or double, or to a left or right sided bed).
      - Attention of MD urgently needed (in case immediate action seems necessary)
  - In case the number and type of patients present is larger than anticipated, it is possible that patients who normally should be in wagon 1 or 2, can be assigned to wagon 3.
- Communicate with allocator and wagon 3 for capacity of train

Allocation

- Who
  - National staff nurse
  - With support of the expat nurse if needed
  - (Logistician)
- What
  - Present in front of the train and await patients on stretchers or wheelchairs
  - Assign a bed number to each presenting patient, taking into account the position in the train (front or back), preferred accessibility to the patient on the left or right, need of a double bed, sex, accompanying caretakers, available beds and the queue of each wagon. Also keep into account only in the first wagon there is a possibility for suctioning.
  - Communicate to Triage MD by radio if nearing full occupancy of wagons.
  - Logistician helps in movement of patients when required

Wagon 1 (Red) or 2 (Yellow)

- Who (minimum)
  - One nurse per wagon
  - One nurse aid per wagon
  - One MD for both wagons
  - (Logistician)
- What
  - Put wheelchairs and stretchers outside the wagons, install ramp to wagons if low platform.
  - Nurse aid manages the flow of stretchers in and out of the wagon.
  - Nurse helps to install patients in their bed. Calls doctor if unstable patient

© 2023 Walravens S et al. *JAMA Network Open.*

- MD supervises both wagons and starts treating patients if unstable

Wagon 3 (Green)

- Who
  - Nurse aids
- What
  - Management of flow of patients and caretakers referred to wagon 3, ensuring patients are allocated to the side closest to wagon 2.
  - Nurse aid, with radio, keeps overview of total amount of patients in their wagon
  - When requested by Triage MD on radio, assist in transfer of patients from triage to their wagon with wheelchair. One person should always remain at wagon 3.

Project Coordinator – translator

- What
  - Communicates with authorities on arrival.
  - If no longer needed will assist in regulation of the different wagons according to need.

Plan B

In case there are no ambulance personnel present and the patients are not mobile, the patients will need to be carried in by the help of MSF staff. If this is the case, the MD responsible for triage will announce Plan B over the radio. The allocator and responsible for the 3rd wagon will then announce Plan B to all the wagons.

Previously appointed staff will then go outside to present to the Triage area. These will normally be the logistician, both the nurse aids of wagon 1 and 2, and 2 staff members of wagon 3. At any moment 2 nurses and 1 doctor should remain present in wagon 1 and 2, and one person in wagon 3.

The staff outside will then be sent with a patient to the allocator to go further to wagon 1 or 2, or will be directly send to wagon 3.

Communication

The responsible for triage, for allocation and for wagon 3 will all have a radio to communicate. Communication needs to be kept clear and short.

Security

Whenever MSF moves to a volatile region, every staff member will be equipped with a blast vest and a helmet. The vest can be worn whenever the staff member would like to or obligatory on order of the Project Coordinator. It should be worn under other cloths. The helmet can only be worn after permission or on order of the Project Coordinator as we don't want to cause unnecessary fear with the population.

If, while being in the railway station, the Project Coordinator gives the order "Abort" (during embarkment) or "Cover", everyone should directly hide under the train. If carrying a patient at this moment, you must try to ensure the safety of this patient first by bringing him along or by returning him to the vehicle he came from.

Abbreviations: MSF – Médecins Sans Frontières (Doctors without Borders), MD – Medical Doctor, ER – Emergency Room

© 2023 Walravens S et al. *JAMA Network Open.*

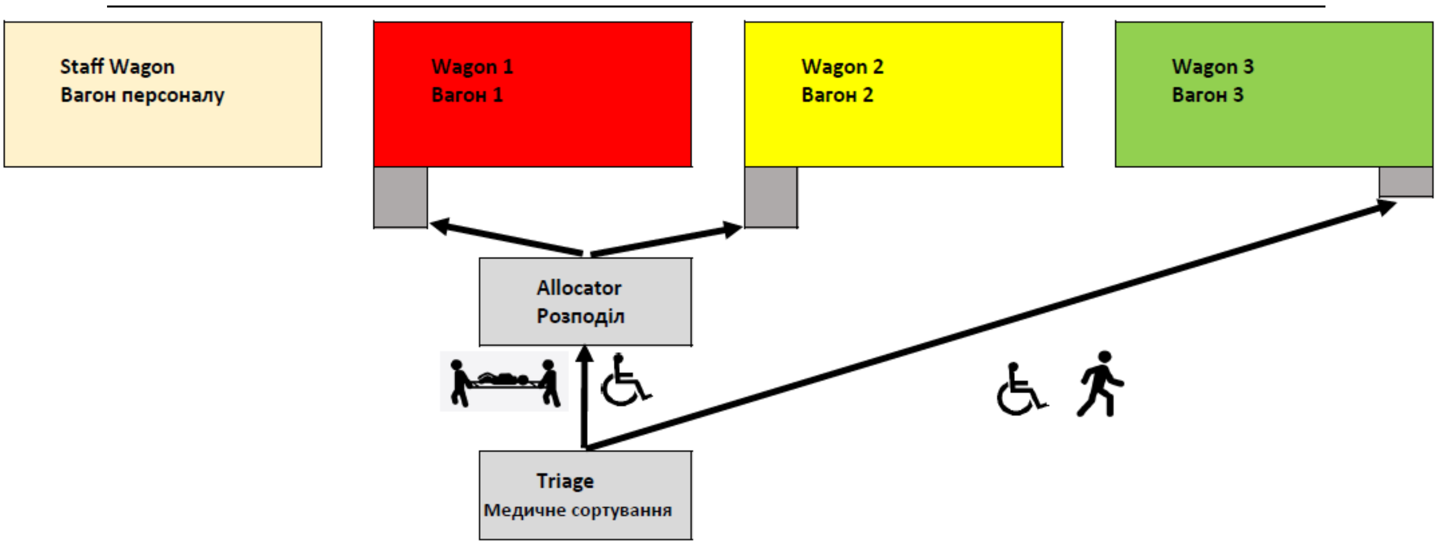

Посадка на базовий медичний потяг

Вступ

Структурована та швидка посадка на потяг є важливою, аби створити умови для оптимального догляду за нашими пацієнтами та забезпечити максимальну безпеку. *Структурованість* допомагає відокремити більш важких пацієнтів від менш важких, полегшити медсестринську допомогу та оптимізувати комфорт пацієнтів. *Швидкість* дозволяє дотримуватися запланованого графіку та мінімізувати час перебування на платформі в нестабільних регіонах.

Хоча ми щоразу плануємо посадку заздалегідь, складні та неочікувані ситуації все одно можуть статися. У найбільш стандартній ситуації всі швидкі стоять на платформі в ряд, а клінічний статус кожного пацієнта відомий. Відхиленнями він стандартного сценарію, які вже ставалися чи можуть статися, є ситуації, коли швидкі прибувають по черзі, немобільні постраждалі приїжджають на автобусах, пацієнти чекають наодинці без супроводу медпрацівників, прибуває більше пацієнтів або у більш критичному стані, ніж очікувалося, стан пацієнтів занадто нестабільний для перевезення або ж посадку доводиться перервати чи пришвидшити з міркувань безпеки тощо.

Стандартний план або План А описаний нижче. За зміни обставин необхідно адаптуватися до нової ситуації та за потреби повідомити певних членів команди. Якщо на вокзалі немає персоналу швидкої медичної допомоги, аби занести пацієнтів до потяга, ми працюємо за Планом Б, згідно якого більшість членів команди MSF виходить з вагонів на платформу, аби перемістити пацієнтів на ношах та візках до вагонів.

План А - Зони та ролі

Перед кожною посадкою пацієнтів, координатор потяга та/або іноземний лікар швидкої допомоги і екстреної медицини збирає зустріч, щоби повідомити членів команди про приблизну кількість пацієнтів, їх тяжкість та очікувану безпекову ситуацію на станції. Таким чином, відбувається ознайомлення персоналу з планом дій та розподіл ролей. Також повідомляються можливі відхилення від стандартного плану.

© 2023 Walravens S et al. *JAMA Network Open.*

Медичне сортування

- Хто
  - Українські лікарі
  - Та за необхідності іноземний лікар швидкої допомоги і екстреної медицини
- Що
  - Оцінюють безпеку та план дій. Адаптують план, якщо це необхідно, та інформують команду.
    - Оголошують про перехід на ПЛАН Б по рації у випадку, якщо потрібні додаткові помічники для перенесення пацієнтів.
  - Якщо пацієнтів супроводжують доглядальники, необхідно представитися та пояснити їм план дій.
  - Визначають зону медичного сортування та розподілу між вагонами 1 і 2.
  - Регулюють заїзд та виїзд швидких. Забезпечують організовану роботу.
  - Відокремлюють пацієнтів вагону 1 та 2 від пацієнтів вагону 3.
    - Вагон 3 або ЗЕЛЕНИЙ вагон призначений для відносно мобільних пацієнтів, які можуть самостійно ходити до вбиральні, потребують мінімальної медсестринської допомоги та можуть самі приймати призначені їм медикаменти.
      - Такі пацієнти прибувають до вагону 3 пішки або на візку. Відповідальний за вагон 3 може надіслати помічника з візком, отримавши відповідний запит по рації.
    - Вагон 1 (червоний) та вагон 2 (жовтий) призначені для немобільних пацієнтів, яких переміщають на ношах та які потребують періодичного чи постійного медсестринського догляду або допомоги в отриманні медикаментів.
      - Таких пацієнтів надсилають на ношах до відповідального за розподіл між вагонами 1 і 2 перед входами у ці два вагони, який призначить ліжко.
      - За необхідності кожен пацієнт отримує записку з такою інформацією:
        - Розміщення спереду (пацієнтам у критичному стані призначають ліжка у передньому краю вагона, аби вони були ближче до поста медсестри).
        - Місце поранення - сторона для дренажу та зовнішніх фіксаторів (впливає на призначення ліжка: односпального чи двоспального, з правої чи з лівої сторони).
        - Термінова допомога лікаря (у разі необхідності негайних дій)
    - Якщо тип пацієнтів інакший або їхня кількість більша, ніж передбачалося, вони можуть зайняти вагон 3 замість стандартних для них вагонів 1 і 2.
  - Спілкуються з відповідальним за розподіл між вагонами 1 і 2 та з вагоном 3 щодо доступних місць.

Розподіл

- Хто
  - Українська медсестра
  - Та за необхідності іноземна медсестра
  - (Логіст)
- Що

© 2023 Walravens S et al. *JAMA Network Open.*

- Очікують перед поїздом і приймають пацієнтів на ношах або на інвалідному візку.
- Призначають кожному пацієнту номер ліжка, враховуючи положення в поїзді (спереду або ззаду), бажаний доступ до пацієнта зліва або справа, потребу в двоспальному ліжку, стать, супроводжуючих доглядачів, наявність вільних ліжок та чергу з ношами до вагона. Також потрібно враховувати, що відсмоктувач є лише в першому вагоні.
- Якщо майже всі місця зайняті, повідомте про це по рації лікаря, відповідального за медичне сортування.
- За потреби логіст допомагає з переміщенням пацієнтів.

Вагон 1 (червоний) або 2 (жовтий)

- Хто (мінімум)
  - Одна медсестра на вагон
  - Один помічник медсестри на вагон
  - Один лікар на обидва вагони
  - (Логіст)
- Що
  - Розміщують інвалідні візки та ноші поза вагонами, встановлюють рампи, якщо платформа занизька.
  - Помічник медсестри контролює використання ношів всередині та на виході з вагону.
  - Медсестра допомагає вкласти пацієнтів на їхні ліжка та викликає лікаря, якщо стан пацієнта нестабільний.
  - Лікар відповідає за обидва вагони і розпочинає лікування пацієнтів, якщо вони нестабільні.

Вагон 3 (зелений)

- Хто
  - Помічники медсестер
- Що
  - Скеровують потік пацієнтів та доглядальників, яких розподілили до вагону 3, призначаючи їм місця якнайближче до вагону 2.
  - Помічник медсестри з рацією слідкує за кількістю пацієнтів у своєму вагоні.
  - Отримавши запит по рації від лікаря, відповідального за медичне сортування, допомагає довезти пацієнтів на візку із зони сортування до вагона. У вагоні 3 завжди повинен залишатися один медпрацівник.

Координатор потяга – перекладач

- Що
  - Спілкуються з органами влади по прибуттю.
  - Після закінчення спілкування, допомагають в регулюванні різних вагонів відповідно до потреби.

План Б

У разі відсутності персоналу швидкої допомоги, немобільних пацієнтів доставляють до вагонів співробітники MSF. У такому випадку лікар, відповідальний за медичне сортування, оголошує про план Б по рації. Відповідальний за розподіл між 1 і 2 вагонами та відповідальний за вагон 3 оголошують про План Б усім співробітникам цих вагонів.

<u>Після цього попередньо визначені співробітники виходять з потяга і підходять до зони медичного сортування</u>. Зазвичай це логіст, помічники медсестер вагонів 1 та 2, а також двоє співробітників

© 2023 Walravens S et al. *JAMA Network Open.*

вагона 3. 2 медсестри та 1 лікар повинні завжди залишатися у вагоні 1 та 2, а одна людина — у вагоні 3.

Співробітників, які вийши з вагонів, спрямовують з пацієнтами до зони розподілу між вагонами 1 і 2 або прямо до вагона 3.

Комунікація
Відповідальні за медичне сортування, розподіл між вагонами 1 і 2 та відповідальний за вагон 3 матимуть рації. Комунікація повинна бути короткою та чіткою.

Безпека
Щоразу, коли MSF прибуває в нестабільний регіон, кожен співробітник отримує бронежилет і шолом. Жилет можна носити щоразу, коли цього побажає співробітник, або ж обов'язково надягати його за вказівкою координатора потяга. Жилет потрібно носити під одягом. Шолом можна носити лише після отримання дозволу або за вказівкою координатора потяга, аби не викликати зайвий страх у населення.

Якщо, перебуваючи на залізничному вокзалі, координатор потяга дає вказівку «Abort/Стоп» (під час посадки) або «Cover/Укриття», всі повинні сховатися прямо під поїздом. Якщо в цей момент ви переносите пацієнта, ви повинні спробувати спочатку подбати про його безпеку, взявши його з собою або повернувши в транспортний засіб, яким він прибув.

© 2023 Walravens S et al. *JAMA Network Open.*

eAppendix 4. Embarkment of advanced medical evacuation train MSF – Version May 2022

<u>Embarkment of advanced medical evacuation train</u>

Introduction

The importance of a structured and fast embarkment of the train is essential to give the optimal care to our patients and to ensure maximal safety. *Structured* to separate the more severe patients from the less severe, to facilitate nursing care and to optimize patient comfort. *Fast* to remain within the planned time schedule and to minimize the time exposed on the platform in volatile areas.

Although we plan every embarkment before arrival, difficult and unexpected situation are likely to occur. The most standard situation is where all ambulances are lined up and the clinical status of every patient is known. Deviations of this situation that have happened or can happen are ambulance coming one by one, busses with non-mobile victims, patients left behind without healthcare workers present, more patients or more severe patients than anticipated, patients who are too unstable for transfer, abortion or speeding up of embarkation due to security issues,…

The standard plan or <u>Plan A</u> is described below. Whenever a different situation occurs it is needed to adapt each time to the situation and wherever needed the correct team members need to be informed.
In case there is no ambulance staff present to transfer the patients into the train, we switch to <u>Plan B</u> in which a large part of the MSF staff in the wagons is send outside to carry the patients on stretchers and wheelchairs into the wagons.

Compared to the embarkment of the basic medical evacuation train, the embarkment of the advanced medical evacuation train has the advantage that beds are more uniform in each wagon and more place is available, making it easier to transfer the patients and making the role of general allocator unnecessary. As a disadvantage the wagons are further from each other, increasing the need for good communication, and we will have more severely ill patients in the ICU, necessitating more direct medical and nursing care on arrival.

Plan A - Zones and roles

Before every embarkment of patients, a meeting will be called by the Project Coordinator, the expat ER MD, or the MD responsible for Triage to announce the estimated number and severity of patients and the anticipated situation in the railway station. Subsequently, the plan for the embarkment will be shared and all staff will be reminded of their role. Planned deviations of the standard plan can also be announced.

Triage

- Who
  - National staff MD
  - With support of expat ER MD if needed
  - Logistician
- What
  - Prior to arrival study list of expected patients to understand overall patient severity and most likely candidates for ICU wagon.
  - On arrival, assessment of situation for safety and plan of action. Adapt plan where needed and communicate this with the team.

© 2023 Walravens S et al. *JAMA Network Open.*

        - Announce switch to PLAN B by radio in case extra helpers for transfer of patients are needed.
    - If present, introduce yourself to representative of all patients to explain plan
    - Define zone for triage
    - Regulation of entry and exit of ambulances. Ensure smooth flow. According to the place of triage and the constitution of the train platform, decide whether to send patients via ambulance or via stretcher to each wagon.
    - Sorting of ICU (RED), IPD 1 (YELLOW), IPD 2 (BLUE) and IPD 3 (GREEN) patients by quick analysis of the history and clinical situation of the patient (<1 minute), handing of a triage color and referral of the patient/ambulance to the correct wagon.
        - ICU (RED) with 5 positions is reserved for the most severe patients or those who are most likely to deteriorate. This is the only wagon with access to ventilation and vasopressors.
        - IPD 1 (YELLOW) or IPD 2 (BLUE) with each 9 positions per wagon for patients who need to be transferred by stretcher, are not mobile, need intermittent or continuous nursing care, or need help in taking medications.
        - IPD 3 (GREEN) with max 16 low and max 16 high positions can accommodate patients who are sufficiently mobile to enter the wagon on their own, need minimal medical attention or nursing care and can take their own medication. This wagon, together with the polyvalent wagon will also be used by the caretakers of the patients in the other wagons.
    - Communicate with the different wagons for verifying capacity of train or problems encountered.
    - Logistician to be in front of the wagons after instalment of ramps to wagons. Assist with triage for practical tasks when requested.

ICU (RED)

- Who (minimum)
    - One MD
    - One nurse
    - One nurse aid
- What
    - Put out red flag. Follow-up Ramp is being installed by logistician and train staff if necessary
    - MD manages the flow of stretchers in and out of the wagon and decides position of each patient. The first 2 bed positions should be reserved for the patients on mechanical ventilation, or with the highest likelihood of needing respiratory support. He/she is also responsible for a first medical assessment of each patient.
    - Nurse and nurse aid help to install patients in their bed and attach to monitor.
    - Communicate with triage in case of problems
    - Refer caretakers of patients to IPD 3 and polyvalent wagon after installation of patient

IPD 1 (YELLOW) or 2 (BLUE)

- Who (minimum)
    - One nurse per wagon
    - One nurse aid per wagon
- What
    - Put out yellow or blue flag. Follow-up Ramp is being installed by logistician and train staff if necessary.

© 2023 Walravens S et al. *JAMA Network Open.*

    - Nurse manages the flow of stretchers in and out of the wagon and the position of each patient.
    - Nurse and nurse aid help to install patients in their bed. Calls doctor in ICU if unstable patient
    - Communicate with triage in case of problems
    - Refer caretakers of patients to IPD 3 and polyvalent wagon after installation of patient

IPD 3 and polyvalent wagon (Green)

- Who
    - Nurse aid(s)
- What
    - Put out green flag
    - Management of flow of patients and caretakers referred to IPD 3 and polyvalent room. Prioritize patients to IPD 3 and caretakers to polyvalent wagon. If remaining positions available in the IPD 3 after embarking, caretakers can make use of these beds.
    - Communicate with triage in case of problems

Project Coordinator – translator

- What
    - Communicates with authorities on arrival.
    - If no longer needed will assist in regulation of the different wagons according to need.

Plan B

In case there are no ambulance personnel present and the patients are not mobile, the patients will need to be carried in by the help of MSF staff. If this is the case, the MD responsible for triage will announce Plan B over the radio and this message should be further spread over the wagons.

<u>Previously appointed staff will then go outside to present to the Triage area</u>. The staff remaining in the wagons is reduced to one or two persons.

Communication

The responsible for triage, for ICU and for IPD 2 will all have a radio to communicate. Communication needs to be kept clear and short.

Security

Whenever MSF moves to a volatile region, every staff member will be equipped with a blast vest and a helmet. The vest can be worn whenever the staff member would like to or obligatory on order of the Project Coordinator. It should be worn under other cloths. The helmet can only be worn after permission or on order of the Project Coordinator as we don’t want to cause unnecessary fear with the population.

If, while being in the railway station, the Project Coordinator gives the order “Abort” (during embarkment) or “Cover”, everyone should directly hide under the train. If carrying a patient at this moment, you must try to ensure the safety of this patient first by bringing him along or by returning him to the vehicle he came from.

<u>Abbreviations</u>: MSF – Médecins Sans Frontières (Doctors without Borders), MD – Medical Doctor, ER – Emergency Room, ICU – Intensive Care Unit, IPD – Inpatient department

© 2023 Walravens S et al. *JAMA Network Open.*

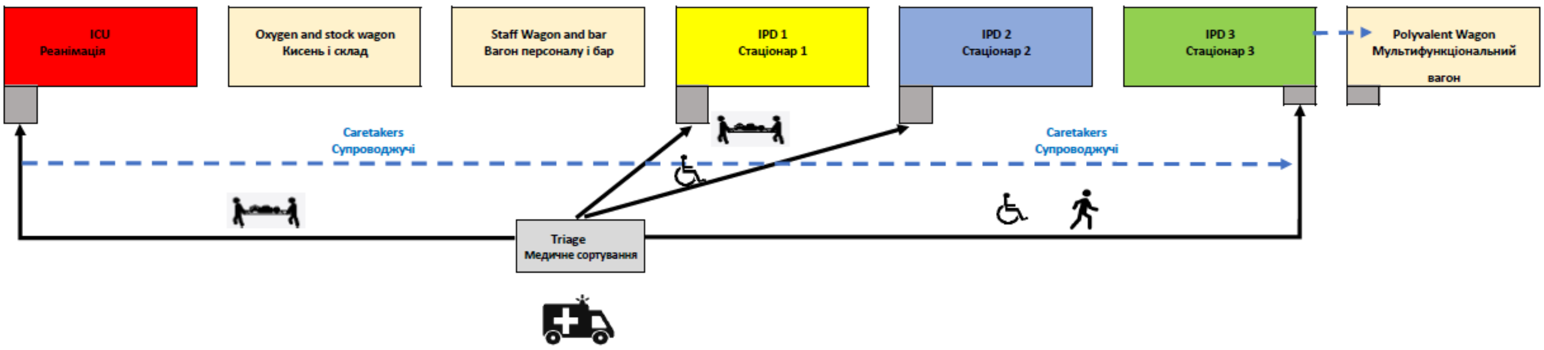

<u>Посадка на спеціалізований медичний евакуаційний потяг</u>

Вступ

Структурована та швидка посадка на потяг є важливою, аби створити умови для оптимального догляду за нашими пацієнтами та забезпечити максимальну безпеку. *Структурованість* допомагає відокремити більш важких пацієнтів від менш важких, полегшити медсестринську допомогу та оптимізувати комфорт пацієнтів. *Швидкість* дозволяє дотримуватися запланованого графіку та мінімізувати час перебування на платформі в нестабільних регіонах.

Хоча ми щоразу плануємо посадку заздалегідь, складні та неочікувані ситуації все одно можуть статися. У найбільш стандартній ситуації всі швидкі стоять на платформі в ряд, а клінічний статус кожного пацієнта відомий. Відхиленнями він стандартного сценарію, які вже ставалися чи можуть статися, є ситуації, коли швидкі прибувають по черзі, немобільні постраждалі приїжджають на автобусах, пацієнти чекають наодинці без супроводу медпрацівників, прибуває більше пацієнтів або у більш критичному стані, ніж очікувалося, стан пацієнтів занадто нестабільний для перевезення або ж посадку доводиться перервати чи пришвидшити з міркувань безпеки тощо.

Стандартний план або <u>План А</u> описаний нижче. За зміни обставин необхідно адаптуватися до нової ситуації та за потреби повідомити певних членів команди. Якщо на вокзалі немає персоналу швидкої медичної допомоги, аби занести пацієнтів до потяга, ми працюємо за <u>Планом Б</u>, згідно якого більшість членів команди MSF виходить з вагонів на платформу, аби перемістити пацієнтів на ношах та візках до вагонів.

Порівняно з посадкою на базовий медичний евакуаційний потяг, посадка на спеціалізований медичний евакуаційний потяг має перевагу, оскільки ліжка у кожному вагоні більш уніфіковані, а вільного місця більше, що робить перенесення пацієнтів легшим, а розподіл між вагонами 1 і 2 непотрібним. Недолік, проте, полягає в тому, що вагони розташовані далі один від одного, що збільшує потребу в якісній комунікації, а також у наявності у вагоні-реанімації більшої кількості тяжкохворих пацієнтів, які потребуватимуть прямого лікарського та медсестринського догляду після прибуття.

План А – Зони та ролі

Перед кожною посадкою пацієнтів, координатор потяга, іноземний лікар швидкої допомоги і екстреної медицини, чи лікар, відповідальний за медичне сортування, збирає зустріч, щоби повідомити членів команди про приблизну кількість пацієнтів, їх тяжкість та очікувану безпекову

© 2023 Walravens S et al. *JAMA Network Open.*

ситуацію на станції. Таким чином, відбувається ознайомлення персоналу з планом дій та розподіл ролей. Також повідомляються можливі відхилення від стандартного плану.

Медичне сортування

- Хто
  - Українські лікарі
  - Та за необхідності іноземний лікар швидкої допомоги і екстреної медицини
  - Логіст
- Що
  - Перед прибуттям ознайомлюються зі списком очікуваних пацієнтів, щоб зрозуміти, наскільки тяжкий їх стан та хто з них скоріше за все буде розміщений у вагоні-реанімації.
  - Оцінюють безпеку та план дій по прибуттю. Адаптують план, якщо це необхідно, та інформують команду.
    - Оголошують про перехід на ПЛАН Б по рації у випадку, якщо потрібні додаткові помічники для перенесення пацієнтів.
  - Якщо пацієнтів супроводжують доглядальники, необхідно представитися та пояснити їм план дій.
  - Визначають зону медичного сортування.
  - Регулюють заїзд та виїзд швидких. Забезпечують організовану роботу. Зважаючи на розміщення зони медичного сортування та особливості платформи, приймають рішення надіслати пацієнтів до вагонів машиною швидкої допомоги чи на ношах.
  - Сортують пацієнтів між реанімацією (ЧЕРВОНИЙ), стаціонаром 1 (ЖОВТИЙ), стаціонаром 2 (СИНІЙ) чи стаціонаром 3 (ЗЕЛЕНИЙ), провівши швидкий аналіз історії хвороби та клінічного стану пацієнта (<1 хвилини), та видають належний колір, щоб спрямувати пацієнта/швидку допомогу до відповідного вагона.
    - Реанімація (ЧЕРВОНИЙ) на 5 ліжок призначена для найтяжчих пацієнтів або тих, чий стан скоріше за все може погіршитися. Це єдиний вагон з доступом до вентиляції та вазопресcорів.
    - Стаціонар 1 (ЖОВТИЙ) чи стаціонар 2 (СИНІЙ), кожний на 9 ліжок, призначені для немобільних пацієнтів, яких переміщають на ношах та які потребують періодичного чи постійного медсестринського догляду або допомоги в отриманні медикаментів.
    - Стаціонар 3 (ЗЕЛЕНИЙ) на максимум 16 нижніх та 16 верхніх ліжок може вмістити пацієнтів, які достатньо мобільні, аби зайти до вагона самостійно, потребують мінімального лікарського чи медсестринського догляду і можуть самостійно приймати призначені їм медикаменти. Цей вагон разом з мультифункціональним вагоном також буде використовуватися доглядачами пацієнтів, розміщених в інших вагонах.
  - Спілкуються з іншими вагонами щодо місткості потяга та у разі виникнення проблем.
  - Після встановлення рамп, логіст залишається перед вагонами та за потреби допомагає зоні медичного сортування, виконуючи практичні завдання.

Реанімація (ЧЕРВОНИЙ)

- Хто (мінімум)
  - Один лікар

- Одна медсестра
- Один помічник медсестри

- Що
  - Виставляють червоний прапорець. Слідкують за тим, чи встановили логіст та провідники рампу, якщо це необхідно.
  - Лікар контролює використання ношів всередині та на виході з вагону і визначає розміщення кожного пацієнта. Перші два ліжка мають бути зарезервовані для пацієнтів, які перебувають на штучній вентиляції легень, абонайбільш імовірно можуть потребувати респіраторної підтримки. Він/вона також відповідає за первинний лікарський огляд кожного пацієнта.

  - Медсестра та помічник медсестри допомагають вкласти пацієнтів до їхніх ліжок та підключити їх до моніторів.
  - У разі виникнення проблем, повідомляють про них зоні медичного сортування.
  - Після розміщення пацієнтів, спрямовують їхніх доглядальників до стаціонару 3 та мультифункціонального вагона.

Стаціонар 1 (ЖОВТИЙ) чи 2 (СИНІЙ)

- Хто (мінімум)
  - Одна медсестра на вагон
  - Один помічник медсестри на вагон
- Що
  - Виставляють жовтий чи синій прапорець. Слідкують за тим, чи встановили логіст та провідники рампу, якщо це необхідно.
  - Медсестра контролює використання ношів всередині та на виході з вагону та визначає розміщення кожного пацієнта.
  - Медсестра та помічник медсестри допомагають вкласти пацієнтів на їхні ліжка та викликають лікаря до реанімації, якщо стан пацієнта нестабільний.
  - У разі виникнення проблем, повідомляють про них зоні медичного сортування.
  - Після розміщення пацієнтів, спрямовують їхніх доглядальників до стаціонару 3 та мультифункціонального вагона.

Стаціонар 3 та мультифункціональний вагон (ЗЕЛЕНИЙ)

- Хто
  - Помічники медсестер
- Що
  - Виставляють зелений прапорець.
  - Спрямовують пацієнтів та супроводжуючих доглядальників до стаціонару 3 та мультифункціонального вагона. При цьому у стаціонарі 3 пріоритет надається пацієнтам, а доглядальників розміщують у мультифункціональному вагоні. Якщо після посадки пацієнтів у стаціонарі 3 залишаються вільні місця, доглядальники можуть зайняти їх.
  - У разі виникнення проблем, повідомляють про них зоні медичного сортування.

Координатор потяга – перекладач

- Що
  - Спілкуються з органами влади по прибуттю.
  - Після закінчення спілкування, допомагають в регулюванні різних вагонів відповідно до потреби.

© 2023 Walravens S et al. *JAMA Network Open.*

План Б
У разі відсутності персоналу швидкої допомоги, немобільних пацієнтів доставляють до вагонів співробітники MSF. У такому випадку лікар, відповідальний за медичне сортування, оголошує про план Б по рації. Це повідомлення необхідно поширити серед персоналу далі по вагонах.

Попередньо визначені медпрацівники виходять з потяга і підходять до зони медичного сортування. Всередині вагонів залишаються одна-дві особи.

Комунікація
Відповідальні за медичне сортування, реанімацію та стаціонар 2 матимуть рації. Комунікація повинна бути короткою та чіткою.

Безпека
Щоразу, коли MSF прибуває в нестабільний регіон, кожен співробітник отримує бронежилет і шолом. Жилет можна носити щоразу, коли цього побажає співробітник, або ж обов'язково надягати його за вказівкою координатора потяга. Жилет потрібно носити під одягом. Шолом можна носити лише після отримання дозволу або за вказівкою координатора потяга, аби не викликати зайвий страх у населення.

Якщо, перебуваючи на залізничному вокзалі, координатор потяга дає вказівку «Abort/Стоп» (під час посадки) або «Cover/Укриття», всі повинні сховатися прямо під поїздом. Якщо в цей момент ви переносите пацієнта, ви повинні спробувати спочатку подбати про його безпеку, взявши його з собою або повернувши в транспортний засіб, яким він прибув.

© 2023 Walravens S et al. *JAMA Network Open.*

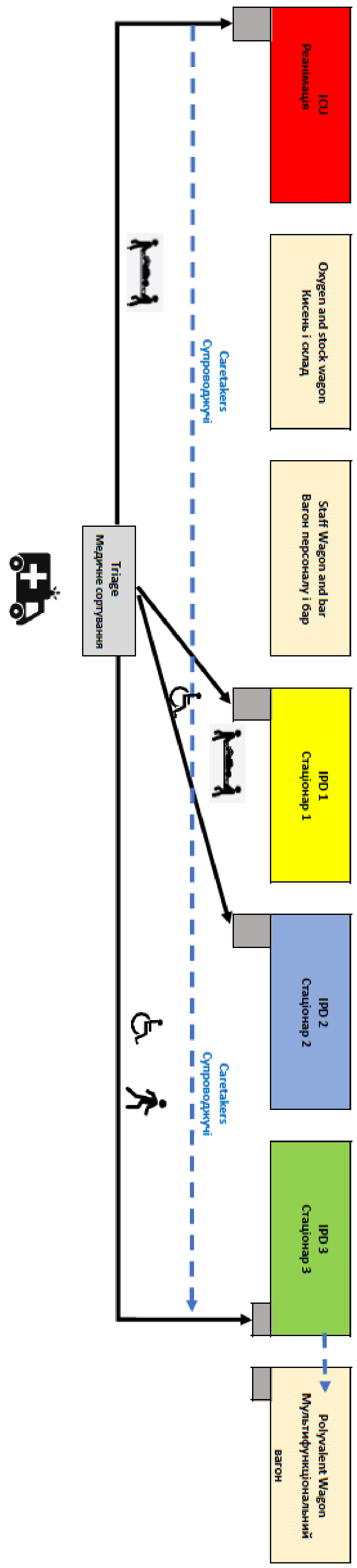

© 2023 Walravens S et al. *JAMA Network Open.*
