# Supplementary material for: Characteristics of Medical Evacuation by Train in Ukraine, 2022
Source: JAMA Netw Open. 2023 Jun 23;6(6):e2319726. doi: 10.1001/jamanetworkopen.2023.19726 (PMC10290241; doi:10.1001/jamanetworkopen.2023.19726)
Supplement: Supplement 2. — Data Sharing Statement [file jamanetwopen-e2319726-s002.pdf]

Data Sharing Statement

Walravens. Characteristics of Medical Evacuation by Train in Ukraine, 2022. *JAMA Netw Open*. Published June 23, 2023. doi:10.1001/jamanetworkopen.2023.19726

Data
Data available: No

Additional Information
Explanation for why data not available: Demographic and medical data were collected in aggregate for routine programmatic purposes. Due to the sensitivity of our work in humanitarian crises and potential security risks, we do not routinely make the data available in a public domain, but can make it available on a case-by-case basis depending on the request. Programmatic materials for implementing a similar medical evacuation project can be shared upon request to the corresponding author.
